# A Primary Adrenal Epithelioid Angiomyolipoma (PEComa) in a Patient with Tuberous Sclerosis Complex: Report of a Case and Review of the Literature

**DOI:** 10.1155/2020/5131736

**Published:** 2020-03-17

**Authors:** Nancy Torres Luna, Jorge Esteban Mosquera, Isin Yagmur Comba, Mustafa Kinaan, Jorge Otoya

**Affiliations:** ^1^Department of Internal Medicine, University of Central Florida College of Medicine, Orlando, FL, USA; ^2^Osceola Regional Medical Center, Kissimmee, FL, USA

## Abstract

Epithelioid angiomyolipomas (EAMLs) are mesenchymal tumors that are part of the family of the perivascular epithelioid cell neoplasms (PEComas). These tumors portray a potential aggressive behavior with metastatic lesions found in around 30% of reported cases. EAMLs might present sporadically or in association with the tuberous sclerosis complex (TSC). They typically involve the kidneys, liver, and lungs. It is extremely rare for these tumors to arise from other organs. The present report describes an unusual case of an adult patient with a history of TSC who developed EAML of the adrenal gland. Moreover, he presented with metastatic disease to the liver, a feature rarely described. The diagnosis of EAMLs can be challenging as they are hard to distinguish from other adrenal or renal tumors without a thorough histopathologic and immunohistochemical evaluation. Due to the potential aggressive behavior of these malignancies, timely diagnosis is extremely important and has significant therapeutic and prognostic implications.

## 1. Introduction

Epithelioid angiomyolipomas (EAMLs) are rare, mesenchymal tumors that belong to the perivascular epithelioid cell neoplasms (PEComas). They also share some histologic features of angiomyolipomas (AMLs), but they are mainly composed of epithelioid cells and lack the typical fat tissue component. While AMLs are usually benign, EAMLs tend to be larger in size and can be malignant. They usually involve the kidneys, liver, and lungs. Therefore, involvement of other organs poses a diagnostic challenge [1–4]. While sporadic PEComa family tumors are extremely rare, their occurrence is higher in patients with tuberous sclerosis complex (TSC), a rare autosomal dominant disease with incomplete penetrance. TSC is a syndrome leading to the development of multiple tumors in the retina, skin, kidneys, adrenals, lungs, and other organs. The estimated worldwide prevalence of TSC is 1 in 6,000 or 12,000 people [5]. We describe the case of a 32-year-old gentleman with a history of TSC who presented with subacute back pain and a large intraabdominal mass. The patient was diagnosed with a primary epithelioid angiomyolipoma/PEComa of the right adrenal gland with liver metastases which was determined postsurgery via histological and immunohistochemical evaluation. To the best of our knowledge, there are fewer than ten reported cases of EAML arising in the adrenal gland. Moreover, metastasis to the liver from a primary adrenal EAML has rarely been described.

## 2. Case Presentation

A 32-year-old gentleman presented to the emergency department (ED) with a 1-week history of right-sided lower back pain. His medical history was significant for TSC. He endorsed fatigue, unintentional weight loss of around 50 pounds for the last 3 months, and night sweats for the past weeks prior to admission. He denied any preceding trauma, fever, urinary symptoms, hematuria, abdominal pain, or changes in bowel movements. Past surgical history was unremarkable. He is a lifetime non-smoker and denied any alcohol or recreational drug use.

Physical examination revealed multiple facial angiolipomas over the nose and cheeks. No enlarged cervical or supraclavicular lymph nodes were found. Respiratory and cardiovascular exams were unremarkable. The abdomen was soft and nondistended, but the right flank was tender to palpation without rebound or guarding. A palpable mass was noted in the right hemiabdomen. Costovertebral tenderness was absent; however, right paraspinal lumbar tenderness was elicited by body movements.

**Figure 1:**
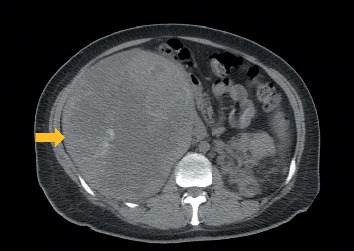
CT abdomen without contrast showing a large, right suprarenal vs. adrenal mass (arrow).

**Figure 2:**
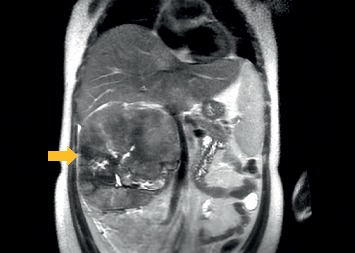
MRI of the abdomen showing a large, right abdominal mass from the unclear origin (arrow).

Laboratory testing was only remarkable for normocytic anemia with hemoglobin 7.8 g/dL (14–18 g/dl). Urinalysis was normal without blood or red blood cells. Computed tomography (CT) scan of the abdomen without contrast revealed a right suprarenal vs. renal mass measuring 16 × 17 × 20 cm (Figure 1). Areas of necrosis, hemorrhage, and parenchymal calcifications were also noted. These findings were confirmed with a magnetic resonance imaging (MRI) study. The origin of this mass (renal vs. adrenal) was indistinguishable on MRI image due to large tumor burden (Figure 2). There were compression and displacement of the inferior vena cava (IVC) medially, but no obvious IVC invasion.

**Figure 3:**
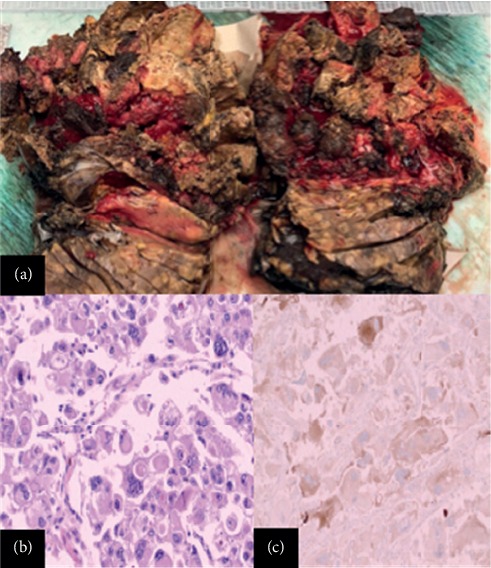
(a) Necrotic mass involving the adrenal gland and perinephric soft tissue. (b) Malignant angiomyolipoma with large tumor cells with abundant eosinophilic cytoplasm. (c) Melan-A/Mart-1 immunohistochemical stain positive within the tumors cells.

Biochemical workup was performed to evaluate whether the mass was of adrenal origin and hormonally active as part of the preoperatory evaluation. Evaluation for metanephrines, normetanephrines, aldosterone, and cortisol overproduction was unremarkable. Subsequently, the patient underwent total right adrenalectomy with en bloc right nephrectomy and resection of regional lymph nodes (Figure 3(a)). Excisional biopsy of segment 5 of the liver was also performed due to intraoperative finding of two liver nodules.

Pathology evaluation showed involvement of the adrenal gland and perinephric soft tissue by malignant, large epithelioid cells with abundant pale to eosinophilic cytoplasm, enlarged and irregular nuclei, and conspicuous nucleoli. These cells are arranged in variably sized nodules with fibrous septae, prominent alveolar growth pattern, with admixed chronic inflammation and extensive necrosis (Figure 3(b)).

Immunohistochemistry showed expression of Melan-A/MART-1 (Figure 3(c)), synaptophysin, HMB-45, and CD10 (focal) by tumor cells, but negative expression of PAX8, CK7, smooth muscle actin, inhibin, chromogranin, calretinin, SOX10, and S100. Ki67 stain revealed an increased proliferative index (up to greater than 50% of tumor cell nuclei staining). The elevated Ki67 proliferative index, cellular atypia, necrosis, and metastatic behavior are consistent with the malignant behavior. The predominance of melanoma markers (Melan-A/MART-1 and HMB-45) along with the absence of adipocytes and predominance of epithelioid cells confirmed the diagnosis of epithelioid angiomyolipoma/malignant PEComa with anaplastic features involving the adrenal gland and perinephric soft tissue. Analysis of the liver biopsies demonstrated a malignant epithelioid neoplasm with the same morphologic features as the adrenal tumor. Given the synchronous nature of these lesions, the liver tumors were considered to be metastatic from the primary adrenal tumor. The patient had no perioperative complications. We discussed with the patient and his family the nature of his malignancy and the high risk of recurrence. The patient opted not to pursue chemotherapy or further surgery even if tumor recurs. He was advised to follow-up with a specialist after his hospital discharge for monitoring and reconsideration of treatment options.

## 3. Discussion

Epithelioid angiomyolipomas (EAMLs) are mesenchymal neoplasms that constitute a variant type of the angiomyolipomas (AMLs), benign tumors composed of adipose tissue, smooth muscle cells, and abnormal thick-walled blood vessels [2–4]. EAMLs differentiate from AMLs as they are mainly composed of epithelioid cells and often lack the typical fat tissue component [6, 7]. They are usually larger in size and have an increased potential for malignant and aggressive behavior. In retrospective studies, overall survival has been calculated to be approximately 1–2 years for advanced/metastatic disease [8, 9]. However, the incidence, prognostic factors, response to treatment, and recurrence of EAMLs have not been clearly determined yet as they are extremely unusual. Moreover, extrarenal presentations of these type of malignancies are even rarer [2]. The average age at diagnosis for AMLs in general is around 40 years old, and the most common initial presentation is focal pain [6, 10, 11]. Case reports of adrenal involvement from AMLs presenting with retroperitoneal hemorrhage after spontaneous rupture have also been described [12].

The diagnosis of EAMLs can be challenging. Clinically, these tumors are usually asymptomatic. As they grow, patients may experience nonspecific symptoms such as weight loss, fatigue, and abdominal discomfort. Although imaging studies can effectively detect the presence of these masses, they have limited utility in distinguishing EAMLs from other neoplasms. Therefore, extensive histopathological and immunohistochemical analyses are necessary to establish the diagnosis.

EAMLs belong to the perivascular epithelioid cell tumors (PEComas) family due to their reaction to immunohistological markers such as SMA and HMB-45 (almost always present), Melan-A (more than 50%), and desmin (around 25%). Histologically, they present as cells arranged in nests, sheets, or alveoli surrounded by septae. These cells can be spindled or epithelioid with clear or eosinophilic cytoplasm, but EAMLs can also present as pleomorphic multinucleated cells [1–3, 7, 13].

Another important clinical feature described in our patient is his history of tuberous sclerosis complex (TSC). EAMLs and AMLs are frequently associated with TSC, with EAMLs presenting more often than AMLs. The incidence of AMLs in general in patients with TSC is 50–90% [7]. A study of 194 cases of renal angiomyolipomas found that 26.7% of patients with renal EAMLs have TSC, while 6.7% with classical AML have a history of TSC [1, 7]. EAMLs are considered to be aggressive malignancies, with approximately one-third of them developing metastatic lesions [4]. Our patient presented liver metastases at the time of the initial diagnosis which were found intraoperatively. As per our literature review, metastasis of EAMLs to the liver from a primary adrenal tumor has not been described before in patients with TSC.

The differentiation between renal and adrenal involvement was challenging in our patient due to the large burden of the mass and was ultimately determined postoperatively. Imaging studies might fail to establish the origin of the tumor, as in our case. EAMLs might share similar features with other malignancies in the imaging studies. Therefore, it is important to perform a thorough evaluation for alternative diagnoses such as hormonally active adrenal tumors prior to proceeding with surgical resection, as these can have implications on perioperative morbidity and mortality. The differential diagnosis considered in our patient included renal cell carcinoma, adrenal cortical carcinoma, hormone-producing adrenal malignancies such as pheochromocytoma and metastatic malignancies such as metastatic melanoma.

Unfortunately, the best treatment approach for malignant EAMLs is not well-established given their rarity, especially extrarenal tumors. There are no clinical trials examining the efficacy of surgical and medical therapies. Management of patients with malignant EAMLs is often guided by findings from cases reported in the literature. Treatment should be individualized based on tumor size, signs of metastasis and progression, and operative risk. While asymptomatic small tumors (<5 cm) can be monitored initially, surgical resection is recommended for most tumors given risk of metastasis, growth, and rupture. However, due to the aggressive behavior of EAMLs, local recurrence, and distant metastasis has been reported after surgery [1, 4].

Recently, focus has been placed on the mTOR pathway with promising results, especially in patients with TSC. TSC is characterized by germ-line mutations in the TSC1 or TSC2 genes which encode the proteins hamartin and tuberin. Tuberin is part of the cell signaling pathway involved in RNA translation (mTOR pathway). Therefore, drugs such as sirolimus or everolimus that suppress mTOR signaling might be potential therapeutic options. Prolonged responses can be achieved with these medications, but costs and side effects are limiting factors [7, 14–17]. Most recently, encouraging results have been published with nanoparticle albumin-bound sirolimus (nab-sirolimus) for treatment of patients with advanced PEComas. Nab-sirolimus is an mTOR inhibitor with higher tumor accumulation. Data presented from a phase 2 trial using nab-sirolimus showed promising results after analysis of disease control rate (DCR), progression-free survival (PFS), and progression-free survival at 6 months (PFS6) with a manageable safety profile, thus representing a treatment option in patients with advanced disease [18]. The use of nivolumab, an anti-PD-1 immune checkpoint inhibitor, has also been described in a patient with malignant renal EAML who had recurrence despite surgery and mTOR inhibitor therapy with everolimus [19]. Additionally, the role of systemic therapy has been explored for this malignancy. A retrospective study evaluated and compared the activity of anthracycline-based and gemcitabine-based chemotherapy, antiangiogenic therapy, and mTOR inhibitors showing a PFS of 3.2 months, 3.4 months, 5.4 months, and 9 months, respectively. These results showed that systemic chemotherapy and antiangiogenic medications might be considered as therapeutic options; however, mTOR inhibitors continue to be considered as the most effective agents [9].

## 4. Conclusion

Primary adrenal epithelioid angiomyolipomas are extremely rare and aggressive malignancies with fewer than 10 cases of primary adrenal EAMLs currently reported in the literature. Histopathology and immunohistochemistry analyses are of utmost importance in establishing the diagnosis of EAMLs. The presence of TSC should also heighten the suspicion of mesenchymal tumors, including malignant EAMLs. Optimal therapeutic strategies, treatment responses, and prognosis are yet to be determined due to the low prevalence of this disease. Further research including clinical trials and prospective studies are necessary to identify and determine potential treatment options that could benefit these patients. Until then, surgical resection remains a mainstay of therapy with mTOR inhibitors as viable options for treatment of recurrence.
